# The impact of chronic diseases on labor participation among rural older adults in China

**DOI:** 10.3389/fpubh.2025.1677445

**Published:** 2025-11-26

**Authors:** FanCun Meng, TianCheng Li

**Affiliations:** 1 School of Public Health, Shantou University, Shantou, Guangdong, China; 2 Guangdong Research Center for Rural Policy, South China Agricultural University, Guangzhou, Guangdong, China

**Keywords:** rural older adults, chronic diseases, labor participation, rural basic medical insurance, mediating effect

## Abstract

**Objective:**

To examine the impact of chronic diseases on labor participation among rural older adults in China and test the mediating role of the New Rural Cooperative Medical Scheme (NRCMS) in this relationship, thereby providing evidence for formulating policies to enhance the health and labor welfare of rural seniors.

**Methods:**

Data from 6,584 rural older adults aged ≥60 years were obtained from the 2023 China Longitudinal Aging Social Survey (CLASS). Statistical analyses were conducted using Stata 17.0 software, including *t*-tests, chi-square tests, and binary logistic regression. Robustness checks were performed using inverse probability weighting regression and by altering the measurement of the independent variable. The mediating effect of the NRCMS was tested using a two-step regression approach.

**Results:**

The prevalence of chronic diseases among rural older adults was 76.6%, and the labor participation rate was 57.9%. After controlling for confounding factors, chronic diseases significantly reduced the risk of labor participation (OR = 0.61, 95%CI: 0.53–0.70). Among specific conditions, cerebrovascular disease had the strongest negative impact (OR = 0.59, 95%CI:0.49–0.71), followed by musculoskeletal disorders (OR = 0.70, 95% CI: 0.61–0.81) and hypertension (OR = 0.77, 95% CI: 0.68–0.87), with all results being statistically significant (*p* < 0.001). Mediation analysis indicated that the NRCMS partially mediated the relationship between chronic diseases and labor participation. This mediating effect showed heterogeneity across income levels and disease burden: it was significant only in the low-income group, and when the number of chronic diseases exceeded three, the proportion of the mediating effect increased to 41.9%.

**Conclusion:**

Chronic diseases constitute a major health risk factor that inhibits labor participation among the rural older adults. The NRCMS plays a significant mediating role in this process. Therefore, targeted measures are recommended, such as strengthening medical insurance coverage and chronic disease management for low-income individuals with multiple comorbidities. These measures would help mitigate the negative impact of health shocks on the labor capacity of the older adults and promote the rational utilization of the older rural workforce.

## Introduction

Population aging presents a global challenge, making the health and labor market participation of older adults a critical issue (1). In developed economies with comprehensive healthcare and pension systems, older individuals often exit the labor force upon experiencing health declines. Conversely, in many developing countries, less mature social safety nets and lower economic resources mean that older adults, particularly in rural areas, frequently must continue working out of economic necessity. China’s context provides a stark illustration of this disparity.

Over half of China’s older adults resides in rural areas, where their labor force participation rates significantly exceed those of their urban counterparts (2). For rural seniors, labor income constitutes a substantial portion (41.2%) of their total income, far surpassing the share in urban areas (22.27%). This phenomenon is largely driven by inadequate rural social security, diminished land-based income, and the weakening of traditional family support systems, compelling many to remain economically active well into old age (3).

Against this backdrop, health status, particularly the burden of chronic non-communicable diseases (NCDs), becomes a pivotal factor influencing labor outcomes (4). In China, approximately 190 million older adults live with NCDs, and about 75% of the older adults is affected by at least one such condition (2, 5). NCDs, characterized by their long duration, slow progression, and association with significant disability, can severely impair functional capacity. For older workers engaged in physically demanding agricultural labor, these health conditions can lead to reduced work efficiency, lower productivity, and poorer performance in the labor market (6, 7).

Therefore, accurately assessing the impact of chronic diseases on labor participation among the rural older adults is crucial for formulating effective responses to population aging. Such research not only aids in helping older adults better manage health risks but also holds significant importance for the scientific development of older human resources, enhancing their self-sufficiency and well-being. Utilizing data from the 2023 China Longitudinal Aging Social Survey (CLASS), this study systematically analyzes the impact of chronic diseases on labor participation among China’s rural older adults and examines the underlying mechanisms, aiming to provide evidence for informed policy-making.

### Theoretical foundations and literature review

The impact of chronic diseases on labor force participation among older adults is primarily explained by two countervailing mechanisms. First, poor health can diminish labor productivity and increase the preference for leisure, leading to labor market withdrawal-a phenomenon akin to a substitution effect. Second, health shocks reduce income and increase medical expenditures, creating a financial imperative to continue working-an income effect. The net effect on an individual’s labor supply depends on the relative strength of these two mechanisms. Health insurance systems, for instance, are generally thought to mitigate the income effect, potentially incentivizing earlier retirement or reduced labor supply. However, the ultimate impact of chronic diseases is not uniform; it varies across populations and is moderated by a range of factors, including the macroeconomic environment, cultural norms, lifestyle, and individual characteristics such as educational attainment (8–10). This heterogeneity precludes universal conclusions about whether chronic diseases increase or decrease labor supply.

Most evidence on the negative health effects on labor participation comes from developed countries, consistently showing that poor health reduces labor force participation among older adults (11, 12). Health status often exerts a stronger influence on retirement decisions than economic factors. Even after addressing the endogeneity of self-reported health, studies find that men in poor health plan to retire 1–2 years earlier. Specific conditions, particularly functional limitations and cardiovascular diseases, are significant drivers of earlier retirement (13, 14). For example, Cai and Kalb (15) using Australian data, found that a decline from good to fair health reduced labor participation by approximately 7% among older adults, a effect far more pronounced than in younger populations. Similarly, Kaiwij and Vermeulen (13) analyzing data from 11 European countries, reported that while non-severe chronic diseases had minimal impact, severe conditions like cancer and heart disease significantly reduced labor participation among individuals aged 50–60 in countries like Australia, Spain, and Denmark.

In contrast to the body of research in developed countries, studies in developing nations, though less abundant, often yield different findings due to distinct economic conditions, social safety nets, and welfare levels. Research in China, for instance, suggests a more complex picture. Benjamin et al. (16), using data from the China Health and Nutrition Survey (CHNS), found that while health deterioration explained 45% of the reduction in labor hours for men aged 60–70, it did not significantly reduce labor force participation rates, indicating that older individuals may need to continue working despite health declines. Significant urban–rural disparities exist within China, reflecting the heterogeneity in the impact of chronic diseases (17–21). Gong et al. (21) found that chronic diseases reduced the probability of labor participation by 46.3% for the “young-old” and 20.6% for the rural older adults. Yang et al. (22), analyzing CHARLS data, demonstrated that the type of health shock matters:chronic conditions like hypertension might reduce working hours without causing complete labor market exit, whereas acute shocks like cancer substantially lower both participation probability and hours worked (23, 24). It is noteworthy that contexts with more developed welfare systems, such as Taiwan, exhibit patterns similar to those in Western countries. Mete and Schultz (25) showed that healthier older adults in Taiwan had significantly higher labor participation rates.

Despite these valuable insights, the existing literature has notable gaps. First, there is a scarcity of studies focusing specifically on the impact of chronic diseases- particularly multiple chronic conditions (multimorbidity)- on the labor participation of the rural older adults in China. Most research in the Chinese context has either focused on specific diseases or general health measures, without deeply exploring the interactions between co-occurring chronic diseases and their compounded effect on labor outcomes (26, 27). Second, while much research has been conducted in developed countries with mature welfare systems, studies from developing countries facing rapid population aging, like China, remain limited. This limits the understanding of how health shocks affect labor supply in settings with less comprehensive pension and insurance systems. Therefore, this study aims to address these gaps by systematically analyzing the impact of single and multiple chronic diseases on the labor participation of China’s rural older adults, with a specific focus on the mediating role of health insurance.

### Research hypotheis

Based on the theoretical analysis and literature review, the following research hypotheses are proposed:

*H1*: Chronic diseases have a significant negative impact on labor participation among rural older adults in China.

The adverse health effects of chronic conditions-such as reduced physical capacity, functional limitations, and increased healthcare needs-diminish older adults’ ability to engage in labor market activities (28–30). Empirical studies confirm that chronic illnesses lower labor force participation, particularly in agricultural and off-farm work, among older adults in both developed and developing contexts (31–33). In rural China, where a substantial proportion of the older adults remain economically active due to limited retirement options and insufficient pension coverage, chronic diseases nevertheless exert a strong depressive effect on labor supply (34–36).

*H2*: Enrollment in health insurance mediates the relationship between chronic diseases and labor participation among rural older adults.

Public health insurance, such as China’s New Rural Cooperative Medical Scheme (NRCMS), can alter the labor supply response to health shocks through two pathways. On the one hand, by reducing out-of-pocket medical expenses and improving access to healthcare, insurance mitigates the financial pressure to work despite illness (34–36). On the other hand, it may encourage preventive health behaviors and better disease management, potentially helping individuals maintain productivity. Studies indicate that health insurance benefits can increase labor participation and reduce the tendency for “ceaseless toil” among the rural middle-aged and older adults (37). Insurance systems thus serve as a key mechanism shaping how health status translates into labor outcomes.

*H3*: The mediating role of health insurance is heterogeneous across income groups and disease severity.

The moderating effect of health insurance is not uniform; it varies with socioeconomic and health conditions. For low-income older adults, medical insurance provides greater financial risk protection, which may be particularly effective in sustaining labor participation when chronic diseases are present (18, 37). However, when the number of chronic conditions exceeds a certain threshold, the mediating effect of insurance becomes more pronounced, as the need for ongoing treatment and income support grows. Such heterogeneity is documented in studies noting that insurance effects are stronger among vulnerable subgroups, including those with multimorbidity or limited economic resources (36).

## Methods

### Data sources

China Longitudinal Aging Social Survey (China Longitudinal Aging Social Survey, abbreviated as CLASS) was designed by the Institute of Gerontology at Renmin University of China and implemented by the China Survey and Data Center at Renmin University of China. It is a national, continuous large-scale social survey project that systematically collects data on the social and economic backgrounds of China’s older adults through regular and systematic data collection. The survey focuses on understanding current older adults care service needs, older adults care resource status, older adults care attitudes and planning, intergenerational relationships, and social adaptation, providing scientific basis for the formulation of reasonable and effective aging policies. The survey employs a stratified multistage probability sampling method, with county-level regions (including counties, county-level cities, and districts) as the primary sampling units and villages/residents’ committees as the secondary sampling units. Within the selected villages/residents’ committees, further sampling of households is conducted, and ultimately one individual aged 60 or older is selected as the survey respondent from each sampled household. The 2023 survey covered 28 provinces/autonomous regions/municipalities in China (excluding Hong Kong, Macao, Taiwan, Hainan, Xinjiang, and Tibet), involving 11,671 older adults across 462 villages/residents’ committees. In accordance with research requirements, this study restricted the research subjects to older adults with rural household registration. After excluding samples with missing, refused, or obviously erroneous key variables, the final valid sample included in the analysis was 6,584 individuals.

### Research design

The theoretical foundation of existing research on labor participation behavior among older adults primarily stems from the Leisure-Work Model (8, 22, 38, 39). First, based on the Leisure-Work Model, we construct the individual utility function *U(C, l)*, where individual utility depends on consumption *(C)* and leisure time *(l)* enjoyed. An individual’s consumption is constrained by their accumulated material wealth and income*ωh* earned from work. Assuming total household wealth is *R*, working hours *h* and leisure hours *l* are constrained by *h + l = T*, where *T* denotes the sum of remaining working and leisure hours after subtracting rest time. Assuming *T* is constant, this transforms into the utility function: *MaxU(C,l),* where *C = R + ωh* and *T = h + l*.

Assuming older adults consumption comprises two components $C_{1}$ and $C_{2}$: $C_{1}$denotes daily consumption with quantity $X_{1}$ and average price $P_{1}$; $C_{2}$denotes healthcare consumption with quantity $X_{2}$ and average price $P_{2}$; then:


$$C=C_{1}+C_{2}=P_{1}X_{1}+P_{2}X_{2}=R+\mathrm{wh}$$ (1)

Assuming *T*, *R*, *ω*, $P_{1}$, and $P_{2}$ are exogenous variables, the labor supply expression can be derived from Equation (1):


$$h=f(R,w,X_{1},X_{2})=(C-R)/w=(C_{1}+C_{2}-R)/w$$ (2)

Equation (2) illustrates the relationship between individual labor supply and health consumption, indicating a theoretical link between labor supply and chronic diseases.

Considering that consumer labor supply behavior correlates with individual demographic characteristics (such as gender, age, marital status, education level, and health status), a set of individual characteristic variables *Z* is introduced to measure these factors. *R* encompasses wealth accumulated during youth and household wealth, which is related to factors such as household demographic structure (40), represented by a set of household characteristic variables *F*. Daily consumption and healthcare expenditures correlate with socioeconomic development levels and social security systems (41), represented by a set of socioeconomic characteristic variables *S*. Thus, the relationship between labor supply and health consumption can be transformed as outlined in Equation 3:


$$L=\alpha_{0}+\alpha_{1}Z+\alpha_{2}F+\alpha_{3}S+\mu$$ (3)

Chronic diseases are a key variable within the set of individual characteristics *Z* that influence labor participation (10, 41). Isolating the chronic disease variable *H* from other individual characteristics, and controlling for other individual, household, and socioeconomic characteristics, the theoretical equation estimating the impact of changes in chronic disease status on labor participation among rural older adults is:


$$L=\alpha_{0}+\mathrm{\beta H}+\alpha_{1}Z+\alpha_{2}F+\alpha_{3}S+\mu$$ (4)

where *L* denotes labor participation status of the older adults, a binary variable where 1 indicates participation and 0 indicates non-participation; *H* represents the chronic disease status of the older adults; *Z* is a set of variables describing individual characteristics of the older adults, including age, gender, marital status, education level, and individual income; *F* is a set of variables describing household characteristics of the older adults, including number of household members and household income; *S* denotes a set of variables describing socioeconomic characteristics, including pension income and home ownership status.

To further examine the mediating role of public health insurance between chronic diseases and labor participation, we extend the model in Equation (4) by introducing the interaction term *H × I* between chronic diseases and enrollment in rural basic medical insurance. The resulting model is presented in Equation 5:


$$L=\alpha_{0}+\mathrm{\beta H}+\mathrm{\theta H}\times I+\alpha_{1}Z+\alpha_{2}F+\alpha_{3}S+\mu$$ (5)

### Measures

#### Dependent variable: labor participation

Labor participation was assessed using data from the CLASS 2023. The variable was constructed based on the question: “What is your current status regarding income-generating work/activities?.” Respondents who reported being actively engaged in such work were coded as 1 (participating), and others as 0 (not participating) (42, 43). To further analyze the impact of chronic diseases on different types of labor, employment was categorized using the question: “What type of work do you currently do?.”Responses were classified as:

Non-agricultural employment: Including roles such as national/enterprise/institutional leaders, professional/technical personnel, general office workers, commercial/service/ manufacturing workers, and self-employed/freelancers.

Agricultural employment: Comprising farmers, herders, and fishermen. The labor participation type variable was coded as: no employment = 0, agricultural employment = 1, non-agricultural employment = 2.

#### Independent variable: chronic disease

Chronic disease status was determined based on the self-reported question: “Which chronic diseases do you have?” from the CLASS survey. The question includes 23 conditions, such as hypertension, heart disease, diabetes, cerebrovascular disease, and cancer. Respondents reporting any of these conditions were classified as having a chronic disease (coded as 1), while those reporting none were coded as 0. Specific chronic diseases-namely hypertension, osteoarthritis, and cerebrovascular disease- were analyzed individually due to their high prevalence and significant impact on functional capacity. To ensure robustness, an indicator for the number of concurrent chronic conditions (comorbidity count) was also used.

### Mediating variable: health insurance enrollment

Enrollment in the New Rural Cooperative Medical Scheme (NRCMS) was used to assess the mediating role of health insurance (44, 45). Enrollment status was determined from the CLASS social security module question: “Are you currently enrolled in the Urban and Rural Residents’ Basic Medical Insurance or the New Rural Cooperative Medical Care (NRCMS)?.” A valid enrollment status in the current year, confirmed by premium payment and system records, was coded as 1; otherwise, it was coded as 0.

### Covariates

Control variables were selected based on established literature and data availability to minimize confounding bias. They were grouped into three categories:

1. Individual characteristics: gender (male = 0, female = 1), age (60–69 = 0, 70–79 = 1, 80 and above = 2), educational attainment (primary school or below = 1, junior high school or above = 2), and the natural logarithm of the previous year’s personal annual income.
2. Household characteristics: marital status (unmarried = 0, married with spouse = 1), living arrangement (living alone = 0, living with family = 1), whether financial support was received from children (no = 0, yes = 1), whether financial support was provided to children (no = 0, yes = 1), number of children (no children = 0, children = 1), housing assets (no housing = 0, one housing unit = 1, two or more housing units = 2), and the logarithm of total household income.
3. Socioeconomic characteristics: enrollment in urban/rural pension insurance (no = 0, yes = 1), accessibility of community healthcare services (measured by presence of village health station: no = 0, yes = 1), and region (western = 1, central = 2, eastern = 3, northeastern = 4).

### Analytical approach

Data preprocessing and analysis were conducted using Stata 17.0. Descriptive statistics were presented as means (± standard deviation) for continuous variables and as proportions (%) for categorical variables. Group differences were analyzed using t-tests and ANOVA. Associations between chronic diseases, NRCMS enrollment, and labor participation were examined using Spearman’s rank correlation. The primary analysis employed a binary logistic regression model to assess the impact of chronic diseases on labor participation. Robustness of the findings was checked using two methods: inverse probability weighting regression and changing the measurement of the independent variable. The mediating effect of NRCMS enrollment was tested using a two-step regression approach: (1) Regressing chronic diseases on the mediator (NRCMS enrollment) to estimate coefficient a; (2) After controlling for the mediating variable, test the direct effect of chronic diseases on labor participation (coefficient c’) and the effect of the mediating variable on labor participation (coefficient b); indirect effect = a × b, total effect = direct effect + indirect effect. Significance of the mediation was assessed using bootstrapping with 1,000 resamples via the R Mediation package. The significance level was set at α = 0.05 for two-tailed test.

## Results

### Descriptive statistics

In 2023, the prevalence of chronic diseases among rural older adults reached 76.6%, with a labor participation rate of 57.9% (see Table 1). From an individual perspective, males accounted for 46.7% of the sample while females constituted 53.3%, showing a slightly higher proportion of women. The age structure exhibits typical aging characteristics, with the younger older adults group aged 60–69 constituting over half. The 0–79 age group accounted for 35.6%, while those aged 80 and above comprised 11.3%. Educational attainment was generally low, with 68.5% having primary school education or below, reflecting the overall low educational level of the rural older adults. Regarding household characteristics, 72.8% of older adults have spouses, while 18.3% live alone. The majority reside with family members, necessitating attention to labor participation barriers among the widowed population. Social security coverage is extensive: the New Rural Cooperative Medical Scheme enrollment rate reaches 87.0%, pension insurance participation stands at 88.3%, and village health station accessibility is as high as 92.7%, aligning with current progress in rural medical facility development. Economic indicators reveal a mean logarithm of individual annual income at 9.87 (standard deviation = 1.23) and a mean logarithm of total household income at 10.52 (standard deviation = 1.45). Regarding housing assets, 65.7% of seniors own one property, 25.4% own two or more properties, and 8.9% lack home ownership. Regarding financial support from children, 56.8% of the older adults receive financial support from their children, while 37.3% still provide financial support to their children. 97.1% of the older adults have children, reflecting a relatively intact rural family network. Regional distribution is relatively balanced, with the western region having the highest proportion at 33.6% and the northeastern region having the lowest at 9.4%.

**Table 1:** Descriptive statistics of variables (*N* = 6,584).

Variable category	Variable name	Assignment description	N/Mean	Proportion/standard deviation
Independent variable	Chronic disease	0 = No	1,542	23.4%
		1 = Yes	5,042	76.6%
Dependent variable	Labor participation	0 = Not participating	2,771	42.1%
		1 = Participating	3,813	57.9%
Mediating variable	New rural cooperative medical scheme enrollment	0 = No	856	13.0%
		1 = Yes	5,728	87.0%
Individual characteristics	Gender	0 = Male	3,074	46.7%
		1 = Female	3,510	53.3%
	Age	1 = 60–69 years old	3,497	53.1%
		2 = 70–79 years old	2,343	35.6%
		3 = 80 years old and above	744	11.3%
	Education	1 = Primary school or below	4,512	68.5%
		2 = Junior high school and above	2,072	31.5%
	Annual personal income	Log, continuous variable	9.87	1.23
Household characteristics	Marital status	0 = No spouse	1,793	27.2%
		1 = Married with spouse	4,791	72.8%
	Housing type	0 = Living alone	1,205	18.3%
		1 = Living with family	5,379	81.7%
	Housing assets	0 = No housing	587	8.9%
		1 = 1 housing unit	4,325	65.7%
		2 = 2 or more housing units	1,672	25.4%
	Financial support from children	0 = No	2,843	43.2%
		1 = Yes	3,741	56.8%
	Providing financial support to children	0 = No	4,126	62.7%
		1 = Yes	2,458	37.3%
	Number of children	0 = No children	193	2.9%
		1 = Children	6,391	97.1%
	Total household income	Log, continuous variable	10.52	1.45
Socioeconomic characteristics	Rural society participation in pension insurance	0 = No	772	11.7%
		1 = Yes	5,812	88.3%
	Accessibility of social medical services	0 = Inaccessible	480	7.3%
		1 = Accessible	6,104	92.7%
	Region	1 = Western	2,215	33.6%
		2 = Central	1987	30.2%
		3 = Eastern	1763	26.8%
		4 = Northeastern	619	9.4%

Comparisons between labor participation groups reveal that chronic diseases exert a pronounced labor-suppressing effect. The prevalence of chronic diseases is significantly higher in the non-labor participation group (χ^2^ = 79.15, *p* < 0.001), confirming chronic diseases as a core health risk factor for labor withdrawal. Structural differences by age and gender reveal an age gap. The working group included 71.9% of older adults aged 60–69, far exceeding the non-working group’s 44.9%. Conversely, those aged 80+ constituted only 2.1% of the working group versus 15.3% of the non-working group, confirming advanced age as a key factor in labor withdrawal. Regarding gender: women constituted 58.7% of the non-labor force participation group and 49.4% of the labor force participation group, indicating rural women are more likely to exit the labor market due to family roles or health issues. Social support exhibited a double-edged sword effect: While health insurance coverage was significantly higher in the labor participation group, 71.4% of those with chronic illnesses remained in the workforce, indicating that insurance failed to fully offset the negative impact of illness on labor capacity. Regarding pension coverage, the labor participation group had a coverage rate of 95.3%, compared to 85.2% in the non-participation group. This suggests that pension security may delay labor withdrawal but cannot fully replace labor income. Family support demonstrated a clear positive effect: 91.4% of those living with family members were in the labor participation group, compared to 77.4% in the non-participation group, proving that family support networks are a crucial foundation for maintaining labor capacity in old age. Regarding economic factors, the labor participation group exhibited significantly higher personal annual income and log mean values than the non-participation group, indicating a close correlation between labor participation and economic needs (see Table 2).

**Table 2:** Comparative analysis of labor participation patterns among rural older adults.

Assignment description		Non-labor force participation (*n* = 2,771, 42.1%)	Labor force participation (*n* = 3,813, 57.9%)	χ^2^/*t* value	*p* value
Chronic disease	No	453 (16.4%)	1,089 (28.6%)	79.15	<0.001
	Yes	2,318 (83.6%)	2,724 (71.4%)		
Rural basic medical insurance enrollment	No	180 (6.5%)	102 (2.7%)	5.87	0.015
	Yes	2,591 (93.5%)	3,711 (97.3%)		
Gender	Male	1,145 (41.3%)	1,929 (50.6%)	75.21	<0.001
	Female	1,626 (58.7%)	1,884 (49.4%)		
Age	60–69 years old	1,842 (44.9%)	1,300 (71.9%)	312.44	<0.001
	70–79 years old	1,632 (39.8%)	470 (26.0%)		
	80 years old and above	630 (15.3%)	39 (2.1%)		
Education	Primary school or below	2,012 (73.4%)	1,040 (57.5%)	42.07	<0.001
	Junior high school and above	759 (26.6%)	769 (42.5%)		
Annual personal income	Logarithm, Mean ± Standard Deviation	9.45 ± 1.18	10.21 ± 1.29	25.67	<0.001
Marital status	No spouse	876 (31.9%)	302 (16.7%)	31.22	<0.001
	Married with spouse	1,895 (68.1%)	1,507 (83.3%)		
Housing type	Living alone	623 (22.6%)	156 (8.6%)	72.18	<0.001
	Living with family	2,148 (77.4%)	1,653 (91.4%)		
Housing assets	No housing	312 (11.3%)	275 (7.2%)	28.45	<0.001
	1 housing unit	1,865 (67.3%)	2,460 (64.5%)		
	2 or more housing units	594 (21.4%)	1,078 (28.3%)		
Financial support for children	No	1,325 (47.8%)	1,518 (39.8%)	15.73	<0.001
	Yes	1,446 (52.2%)	2,295 (60.2%)		
Providing financial support for children	No	1,785 (64.4%)	2,341 (61.4%)	8.92	0.003
	Yes	986 (35.6%)	1,472 (38.6%)		
Number of children	No children	89 (3.2%)	104 (2.7%)	5.28	0.022
	Children present	2,682 (96.8%)	3,709 (97.3%)		
Total household income	Logarithm, Mean ± Standard Deviation	10.28 ± 1.36	10.69 ± 1.51	12.34	<0.001
Rural social pension insurance participation	No	408 (14.8%)	85 (4.7%)	48.62	<0.001
	Yes	2,363 (85.2%)	1,724 (95.3%)		
Access to social medical services	No	226 (8.2%)	65 (5.3%)	9.25	0.002
	Yes	2,545 (91.8%)	1,714 (94.7%)		
Region	Western	987 (35.6%)	1,228 (32.2%)	32.16	<0.001
	Central	852 (30.7%)	1,135 (29.8%)		
	Eastern	689 (24.9%)	1,074 (28.2%)		
	Northeastern	243 (8.8%)	376 (9.9%)		

Further analysis of chronic disease prevalence patterns reveals that hypertension has the highest incidence rate at 32.7%, equivalent to one in every three older adults affected, making it the primary health threat among rural seniors. The prevalence of musculoskeletal diseases stands at 22.5%, while cerebrovascular diseases affect 11.3% of the population. Comorbidity is widespread, with 93.2% of all chronic disease patients experiencing multiple conditions, averaging 3.18 chronic diseases per person. The co-morbidity rates for cerebrovascular disease, chronic lung disease, and heart disease all exceeded 95%, reflecting the close association between cardiovascular-cerebrovascular and respiratory system diseases. Particularly noteworthy is that cerebrovascular disease exhibits characteristics of “high co-morbidity and high burden”: with a co-morbidity rate of 95.6%, patients suffer from an average of 3.92 other diseases, validating its disabling nature and systemic damage to overall health, as shown in Table 3.

**Table 3:** Prevalence of major chronic diseases and comorbidity rates.

Chronic disease type	Number of cases (%)	Comorbidity rate (%)	Average number of comorbidities (*x̄* ± *s*, types)
Hypertension	2,150 (32.7)	1,946 (90.5)	2.87 ± 1.98
Diabetes mellitus	980 (14.9)	928 (94.7)	3.16 ± 2.11
Cerebrovascular disease	745 (11.3)	712 (95.6)	3.92 ± 2.34
Musculoskeletal diseases	1,484 (22.5)	1,380 (93.0)	2.93 ± 2.03
Chronic pulmonary diseases	912 (13.9)	878 (96.3)	3.45 ± 2.28
Heart disease	1,023 (15.5)	981 (95.9)	3.32 ± 2.17
Gastrointestinal diseases	964 (14.6)	906 (94.0)	2.85 ± 1.96
Other chronic diseases	1,803 (27.4)	1,725 (95.6)	3.21 ± 2.25
All chronic disease patients	5,042 (76.6)	4,697 (93.2)	3.18 ± 2.16

### Logistics model

Using labor force participation among rural older adults as the dependent variable and chronic diseases, age, gender, education level, marital status, and rural social pension insurance as independent variables, a binary logistic regression was conducted. The regression results are shown in Table 4. Model fit indicators indicate a −2 Log Likelihood of 5802.71 and a Pseudo R^2^ of 0.195, with the model being statistically significant overall (*p* < 0.001). After controlling for variables such as gender and age, chronic diseases were found to significantly reduce labor participation rates among rural older adults individuals (OR = 0.61, 95% CI: 0.53–0.70). This result aligns with existing research findings, confirming that chronic diseases exert a significant negative impact on labor participation among the older adults (28–30).

**Table 4:** Results of binary logistic regression analysis of chronic diseases and labor participation among rural older adults.

Variable	Model 1 OR (95%CI)	Model 2 OR (95%CI)	Model 3 OR (95%CI)
Chronic diseases	0.61** (0.53–0.70)	–	–
Hypertension	–	0.75** (0.66–0.85)	0.77** (0.68–0.87)
Osteoarthritis	–	0.68** (0.59–0.78)	0.70** (0.61–0.81)
Cerebrovascular disease	–	0.56** (0.47–0.67)	0.59** (0.49–0.71)
Gender (female)	0.47** (0.41–0.54)	0.46** (0.40–0.53)	0.47** (0.41–0.54)
Age (70–79 years)	0.44** (0.38–0.51)	0.43** (0.37–0.50)	0.44** (0.38–0.51)
80 years and older	0.12** (0.09–0.16)	0.11** (0.08–0.15)	0.12** (0.09–0.16)
Educational attainment (junior high school education or above)	1.25** (1.08–1.45)	1.23** (1.06–1.43)	1.22** (1.05–1.42)
Personal annual income	1.08** (1.02–1.14)	1.07** (1.01–1.13)	1.08** (1.02–1.14)
Marital status (married)	1.36** (1.18–1.57)	1.34** (1.16–1.55)	1.33** (1.15–1.54)
Living arrangement (living with family)	1.42** (1.24–1.63)	1.40** (1.22–1.61)	1.38** (1.20–1.59)
Housing assets (1 property)	1.12 (0.98–1.28)	1.11 (0.97–1.27)	1.12 (0.98–1.28)
2 or more properties	1.18* (1.01–1.38)	1.17* (1.00–1.37)	1.18* (1.01–1.38)
Financial support from children	1.15* (1.02–1.30)	1.14* (1.01–1.29)	1.15* (1.02–1.30)
Provides financial support to children	1.06 (0.94–1.20)	1.05 (0.93–1.19)	1.06 (0.94–1.20)
Number of children (with children)	1.09 (0.85–1.40)	1.08 (0.84–1.39)	1.09 (0.85–1.40)
Total household income	1.05 (0.99–1.11)	1.04 (0.98–1.10)	1.05 (0.99–1.11)
Participation in rural social pension insurance	–	–	0.86* (0.74–0.99)
Accessibility of social medical services	–	–	1.21** (1.04–1.41)
Region (central)			1.07 (0.92–1.24)
Eastern			1.16* (1.00–1.35)
Northeastern			1.21* (1.01–1.45)
Model fit indicators			
−2 Log Likelihood	5802.71	5778.63	5755.29
Pseudo R^2^	0.195	0.207	0.218

*p < 0.1, **p < 0.05, ***p < 0.001.

Further analysis of the impact of different chronic diseases revealed: Hypertension reduced the probability of labor participation by 23% (OR = 0.77, 95% CI: 0.68–0.87); musculoskeletal disorders reduced the probability by 30% (OR = 0.70, 95% CI: 0.61–0.81); cerebrovascular disease had the most significant negative impact on labor participation, reducing the probability by 41% (OR = 0.59, 95% CI: 0.49–0.71). Due to its frequent association with motor function impairment, cerebrovascular disease imposes the most pronounced limitations on work capacity.

Regarding controlling variables, the labor participation rate among older adults women was significantly lower than that of men (OR = 0.47, 95% CI: 0.41–0.54); Advanced age markedly reduced labor participation, with the 70–79 age group having a participation rate of only 44% relative to the 60–69 age group (OR = 0.44, 95% CI: 0.38–0.51), and the 80 + age group at just 12% (OR = 0.12, 95% CI: 0.09–0.16). Having a spouse positively promoted labor participation (OR = 1.36, 95% CI: 1.08–1.45). Regarding economic factors, older adults with higher annual personal income exhibited higher labor participation rates (OR = 1.08, 95% CI: 1.02–1.14), indicating that economic needs are a key driver for continued labor among the older adults. Housing assets (owning two or more properties) also showed a positive impact (OR = 1.18, 95% CI: 1.01–1.38), potentially reflecting that older adults with more assets have greater resources to engage in business activities. Financial support from children had a slight positive effect on labor participation (OR = 1.15, 95% CI: 1.02–1.30), possibly related to improved health following the receipt of support. Analysis of socioeconomic characteristics revealed that older adults participating in rural social pension insurance exhibited lower labor force participation rates (OR = 0.86, 95% CI: 0.74–0.99), consistent with labor-leisure model predictions that pension income may increase older adults’ demand for leisure. Access to community healthcare services positively influenced labor participation (OR = 1.21, 95% CI: 1.04–1.41), likely because convenient medical services reduced health risks associated with working while ill. Regional variables indicated that labor participation rates among the older adults were higher in the eastern (OR = 1.16, 95% CI: 1.00 ~ 1.35) and northeastern (OR = 1.21, 95% CI: 1.01–1.45) regions exhibit higher labor participation rates among older adults than the western region, potentially reflecting disparities in regional economic development and employment opportunities. Thus, Hypothesis 1 is validated.

### Nonlinear relationship test

To further validate the impact of the number of chronic diseases on labor participation among rural older adults and examine whether the negative effect accelerates with disease accumulation, a binary logistic regression model incorporating both the first-order and second-order terms of chronic disease count was constructed. The regression results are presented in Table 5.

**Table 5:** Results of the nonlinear relationship between the number of chronic diseases and labor participation among rural older adults.

Variables	β	S. E.	OR	95%CI	*P* value
Number of chronic diseases (first-order term)	−0.215	0.048	0.087	0.734–0.886	<0.001
Number of chronic diseases (second-order term)	−0.041	0.011	0.960	0.940–0.980	<0.001
Control variables	Controlled	Controlled	Controlled	Controlled	Controlled
Constant term	1.892	0.215	6.639	–	<0.001
Model fit metrics					
−2 Log Likelihood	5815.38				
Cox and Snell R^2^	0.187				
Nagelkerke R^2^	0.262				

*p < 0.1, **p < 0.05, ***p < 0.001.

The results indicate that both the coefficient of the linear term and the quadratic term for the number of chronic diseases are negative and highly significant at the 1% level. The linear term coefficient represents the linear effect of the number of chronic diseases on the log-ratio of labor participation among rural older adults. The negative quadratic term coefficient suggests that as the number of chronic diseases increases, the negative impact-specifically the decline in labor participation probability-does not occur at a constant rate but intensifies progressively. In other words, each additional chronic disease erodes the labor capacity of rural older adults more severely than the preceding one, revealing an “accelerating health deterioration” phenomenon. The impact of chronic diseases on labor participation among rural older adults is not a simple linear relationship but exhibits a significant “health deterioration threshold” effect.

### Correlation analysis

Spearman correlation analysis revealed a significant negative correlation between chronic disease prevalence and labor participation (*r* = −0.243, *p* < 0.001). NCHF enrollment status showed a significant positive correlation with labor participation (*r* = 0.314, *p* < 0.001). The prevalence of chronic diseases exhibited a significant positive correlation with NCHF enrollment status (*r* = 0.195, *p* < 0.001) (see Table 6).

**Table 6:** Correlation analysis between chronic diseases, NCHF enrollment status, and labor participation in older adults (r).

Variables	Labor participation	Chronic diseases	NCHF enrollment status
Labor participation	1.000		
Chronic diseases	−0.243***	1.000	
NCHF enrollment status	0.314***	0.195***	1.000

*p < 0.1, **p < 0.05, ***p < 0.001.

### Intermediary mechanisms

The results of the Bootstrap mediation analysis (Table 7) indicate that participation in the New Rural Cooperative Medical Scheme (NRCMS) partially mediates the relationship between chronic diseases and labor force participation among the older adults, accounting for 41.96% of the mediating effect. Specifically: Chronic diseases significantly reduce the probability of rural older adults participating in NRCMS (β = −0.328, *p* < 0.001), likely stemming from two “negative shocks”: First, the financial burden of chronic illnesses makes it difficult for some older adults to sustain premium payments; second, deteriorating health may limit their mobility, reducing both their willingness and ability to complete enrollment and reimbursement procedures. Participation in the NRCMS significantly promotes labor force participation (β = 0.628, *p* < 0.001). By reducing out-of-pocket medical expenses, the program mitigates the economic risks of working while ill, thereby functioning as a form of “labor protection.” This finding reveals a dual mechanism through which chronic diseases influence rural older adults labor participation: one is via affecting NRCMS enrollment (indirect effect), and the other is through the direct impairment of health and work capacity caused by illness (direct effect).

**Table 7:** Analysis of the mediating effect of NRCMS enrollment status on the relationship between chronic diseases and labor participation.

Effect path	β	S. E.	95%CI	*P* value
Total effect (c)	−0.491	0.075	−0.538–0.344	<0.001
Direct effect (c’)	−0.285	0.070	−0.422–0.148	<0.001
Indirect effect (a × b)	−0.206	0.037	−0.278–0.132	<0.001
Chronic disease → Rural medical insurance (a)	−0.328	0.048	−0.422–0.234	<0.001
Rural medical insurance → Labor participation (b)	0.628	0.054	0.522–0.734	<0.001
Proportion of mediating effect	41.96%			

*p < 0.1, **p < 0.05, ***p < 0.001.

A deeper examination of differences in the relationship between chronic diseases and labor participation across income groups can help identify the most vulnerable segments of the population. Further analysis grouped households based on median income (low-income group ≤ median; high-income group > median). The results in Table 8 reveal a clear income gradient in the inhibitory effect of chronic diseases on labor participation among rural older adults: the lower the household income, the greater the negative impact of chronic diseases.

**Table 8:** Heterogeneity analysis of the mediating effects in new rural cooperative medical scheme enrollment status.

Effect path	Low-income group	High-income group
Total effect (c)	−0.491***	−0.232***
Labor participation rate	31.5%	52.8%
Chronic disease → NRCMS enrollment (a)	−0.328***	0.105
NRCMS enrollment → labor participation (b)	0.628***	0.267***
Mediating effect (a × b)	−0.206***	−0.028

*p < 0.1, **p < 0.05, ***p < 0.001.

The impact of chronic diseases on labor participation in rural older adults in the NRCMS is significantly moderated by household income. The negative effect is 2.1 times greater for low-income households than for high-income households, indicating that low-income families are less resilient to the shock of chronic disease. When ill, low-income seniors’ labor participation rate is 21.3 percentage points lower than that of high-income individuals, highlighting the hardship of “working while sick.” For low-income individuals, illness may instead trigger “adverse selection” due to financial constraints preventing renewal of coverage. High-income individuals’ insurance decisions remain unaffected by single health shocks, with chronic diseases primarily manifesting as direct health loss effects. NRCMS enrollment provides universal protection for maintaining labor participation among the older adults, but its marginal utility is nearly 2.4 times higher for low-income individuals. This indicates that the mediating effect of NRCMS enrollment is robust and exclusive to the low-income group, revealing a “the poorer, the more evident” pattern that explains 41.96% of the total effect.

Further analysis confirms the existence of a “health loss threshold” between chronic disease burden and labor participation among rural older adults. Using three chronic diseases as the cutoff point, the sample was divided into low disease burden (≤3 diseases) and high disease burden (>3 diseases) groups for regression analysis. Table 9 shows: When the number of chronic diseases is ≤3, each additional chronic disease reduces the probability of labor participation among rural older adults by 15% (marginal effect = −0.15, *p* < 0.01). When the number of chronic diseases exceeds 3, the marginal effect jumps to −42% (marginal effect = −0.42, *p* < 0.001), with significant statistical difference (*t* = 4.32, *p* < 0.001). This indicates that three chronic diseases represent a critical threshold for rural older adults, beyond which the erosion of their labor capacity accelerates significantly. The mediating effect of NRCMS enrollment exhibited a qualitative shift between groups: in the low disease burden group, NRCMS enrollment accounted for only 18.6% of the mediating effect; in the high disease burden group, this proportion rose sharply to 41.9%. This indicates that for older adults with multiple coexisting diseases (>3), NRCMS enrollment becomes critically important in sustaining labor participation. Accordingly, Hypothesis 2 and Hypothesis 3 are validated.

**Table 9:** Threshold effect analysis of chronic disease burden on labor participation among rural older adults.

Variables / Indicators	Low disease burden group (≤3 conditions)	High disease burden group (>3 conditions)
Sample size	4,812 (73.1%)	1,772 (26.9%)
Labor participation rate	68.5%	31.2%
Marginal effect of chronic disease on labor participation	−0.15** (0.03)	−0.42* (0.05)
Proportion of mediating effect via new rural cooperative medical scheme coverage	18.6%	41.9%
Model Pseudo R^2^	0.12	0.29

*p < 0.1, **p < 0.05, ***p < 0.001.

### Robustness tests

To validate the robustness of the aforementioned results, this study conducted robustness tests based on the baseline regression model using two methods: inverse probability weighting regression and changing the type of independent variables, as detailed in Table 10. First, inverse probability weighting (IPW) was applied for propensity score weighting (PSW), followed by weighted regression analysis. As shown in Column (1), the new model yields results largely consistent with the baseline regression model. Replacing continuous variables. Given that binary variables struggle to adequately reflect chronic disease conditions among the older adults, the binary chronic disease variable was replaced with the number of chronic diseases (a continuous variable). This new variable was then reintroduced into the model for estimation. As shown in Column (2), the new model yields results largely consistent with the baseline regression model.

**Table 10:** Robustness analysis results.

Variables and statistics	IPW (1)	Replace exogenous variable (2)
Chronic diseases	−0.382** (0.117)	
Number of chronic diseases		−0.281** (0.024)
Control variables	Controlled	Controlled
Intercept	6.407** (0.897)	6.954** (0.456)
Sample size	6,584	6,584
Goodness of fit	0.157	0.181

*p < 0.1, **p < 0.05, ***p < 0.001.

### Heterogeneity analysis

Further analysis of the impact of chronic diseases on different labor participation types among rural older adults revealed that the inhibitory effect of chronic diseases was more pronounced in the agricultural employment group: chronic diseases reduced the probability of labor participation by 45% (OR = 0.55) in this group, compared to only a 26% reduction (OR = 0.74) in the non-agricultural employment group, with a significant difference between groups (*p* < 0.001). This may stem from agricultural labor being predominantly physically intensive, demanding higher physical function; chronic diseases directly impair physical strength, endurance, and flexibility, making agricultural workers more likely to exit the labor market. Non-agricultural employment, with lower physical demands, allows some patients to maintain participation by adjusting work intensity, as shown in Table 11.

**Table 11:** Heterogeneous analysis of chronic diseases’ impact on different types of labor participation among rural older adults.

Type of labor participation	Chronic disease OR (95%CI)	Hypertension OR (95%CI)	Osteoarthritis OR (95%CI)	Cerebrovascular disease OR (95%CI)
Agricultural employment	0.55* (0.47–0.64)	0.70* (0.60–0.82)	0.62* (0.52–0.74)	0.51* (0.40–0.65)
Non-agricultural employment	0.74* (0.62–0.88)	0.86* (0.72–1.03)	0.82 ** (0.69–0.98)	0.71 ** (0.55–0.92)

*p < 0.1, **p < 0.05, ***p < 0.001.

## Discussion

This study utilized the 2023 CLASS data and employed statistical tools such as binary Logistic regression and a two-step regression (mediating effect). Based on the leisure-work model, it examined the impact of chronic diseases on labor participation among rural older adults in China and its underlying mechanisms, constituting an important supplement to existing labor participation literature-which predominantly focuses on developed economies like Australia. Drawing from empirical findings, we propose several practical recommendations to address China’s aging challenges.

Current literature lacks consensus on the relationship between chronic diseases and labor force participation among older adults. Some scholars argue for a negative correlation between chronic diseases and labor force participation (8, 46–48), consistent with our findings. Specifically, this study confirms that chronic diseases are a key health risk factor inhibiting labor participation among rural older adults. A significant “health threshold effect” is observed: the negative impact of chronic diseases sharply increases when the number of conditions exceeds three. Chronic diseases substantially prolong the duration of illness and dependence in older adults’ lifetimes, reducing labor participation rates among those capable of working (10). These findings can be attributed to the vital role of public health insurance in improving older adults health outcomes (49), which effectively alleviates medical burdens (50) and enhances healthcare utilization (51). The core finding-chronic diseases significantly inhibit labor participation-aligns with most studies grounded in health human capital theory. Our results further reveal that this impact is not a simple linear relationship but exhibits an accelerated decline with nonlinear characteristics. When the number of chronic diseases exceeds three, the decline in labor participation probability significantly accelerates. This discovery of a “health deterioration threshold” deepens our understanding of the cumulative effects of multimorbidity and suggests that policy interventions should prioritize populations with high disease burden.

Of course, scholars continue to debate the relationship between chronic diseases and labor participation among older adults. Some studies argue for a significant positive correlation (7, 52, 53), while others find no significant association (18, 37, 54), which contradicts our findings to some extent. This discrepancy may stem from chronic diseases being widely recognized as major public health concerns (35, 46, 48, 55), prompting older adults to continue labor participation after retirement to maintain higher activity levels and slow physical decline.

Furthermore, our study reveals heterogeneous impacts of chronic diseases across groups: First, chronic diseases exert a far greater negative impact on agricultural workers than on non-agricultural workers, confirming the high physical demands of agricultural labor (46–48, 56, 57). Second, older age, female gender, and low income make older adults more vulnerable to health shocks, reflecting the interplay of health inequalities with socioeconomic status and gender roles (22, 30, 58, 59). Third, the phenomenon of “working while sick” observed among low-income patients- continuing labor to cover medical expenses-reveals how health shocks may trap older adults in a vicious cycle of “health deterioration-economic pressure-labor depletion” when social safety nets remain inadequate (21, 37, 48).

Regarding the underlying mechanisms, this study reveals a complex and insightful picture. The New Rural Cooperative Medical Scheme (NRCMS) partially mediates the relationship between chronic diseases and labor participation, but its pathway may run counter to conventional expectations. We found that chronic diseases may exert a negative indirect effect by reducing enrollment probability, thereby diminishing labor participation protection. This finding may reflect the “adverse selection” challenge facing rural healthcare systems: older adults with chronic diseases, particularly severe ones, may discontinue coverage due to financial strain, mobility limitations, or insufficient policy understanding, thereby forfeiting the health maintenance and economic risk-sharing benefits of insurance (60–65). This contrasts with Guan et al.’s (48) finding that the New Rural Cooperative Medical Scheme (NRCMS) exerts a positive moderating effect, highlighting the vulnerability of health insurance coverage among low-income and high-disease-burden populations. Furthermore, heterogeneity analysis of the mediating effect indicates that the protective role of NRCMS is concentrated in low-income groups and those with high disease burden, underscoring its importance as a social safety net for the most vulnerable.

Given the negative impact of chronic diseases on labor participation among China’s rural older adults, this study proposes an evidence-based strategy package to mitigate these effects through comprehensive interventions. Recommended strategies include: First, implementing targeted chronic disease management and protection strategies. Establish a tiered management system for older adults chronic diseases based on village clinics, focusing on monitoring, follow-up, and medication guidance for high-risk individuals with three or more chronic conditions. Simultaneously, optimize the New Rural Cooperative Medical Scheme (NRCMS) by exploring measures such as premium reductions for older adults​ with chronic diseases, increasing outpatient reimbursement rates, and expanding the chronic disease medication list to reduce out-of-pocket medical expenses and prevent insurance discontinuation due to illness. Second, promote the development and protection of older adults human resources. Create flexible, light-duty non-agricultural employment opportunities for younger seniors in relatively good health. For agricultural workers, promote age-friendly agricultural tools and techniques to reduce labor intensity. Integrate labor capacity assessments into primary healthcare services to guide older adults in arranging work appropriately based on their health status. Third, strengthen early prevention and health promotion for chronic diseases. Shift the focus of chronic disease screening and health management upstream by conducting health education and early interventions targeting adults aged 55 and above. This approach delays the onset and progression of chronic diseases at their source, serving as a fundamental strategy to maintain labor capacity in later life.

## Conclusion

In conclusion, this study aims to examine the impact of chronic diseases on labor participation among rural older adults in China. Previous research indicates that multiple factors influence labor participation among the older adults (10, 22, 42). Our findings reveal that chronic diseases exert a significant negative effect on labor participation among rural older adults in China, with this impact exhibiting cumulative effects and group heterogeneity. Rural basic medical insurance plays a crucial mediating role, though its effectiveness is constrained by socioeconomic factors and the design of the insurance system. The study also found that the negative impact of chronic diseases is particularly pronounced among those engaged in agricultural labor, low-income individuals, and older women.

It is important to emphasize that this study employs a cross-sectional design, requiring caution when inferring causal relationships between chronic diseases and labor force participation. Due to data limitations, chronic disease measurement relies on self-reported data, which may introduce some error. Future research could utilize longitudinal tracking data combined with clinical diagnostic information to more accurately reveal the dynamic causal relationship between health and labor supply. Additionally, further exploration is warranted into the impact of factors such as chronic disease severity and mental health on labor participation decisions.

## Data Availability

The datasets presented in this article are not readily available because data analyzed in this study were sourced from the China Longitudinal Aging Social Survey (CLASS), a national longitudinal research project designed and implemented by the Institute of Gerontology at Renmin University of China. The author gratefully acknowledges the assistance in data access provided by the CLASS team. Request to access the datasets should be directed to http://class.ruc.edu.cn/.

## References

[1] Shen Y Liu HL. The effect of health services on residence willingness of the floating older adults. Popul Dev. (2022) 2:45–56.

[2] National Health Commission of the People’s Republic of China. (2019). Available online at: http://www.nhc.gov.cn/xcs/s7847/201907.shtm (Accessed February 22, 2023).

[3] Pang L de Brauw A Rozelle S. Working until you drop:the elderly of rural China. China J. (2004) 52:73–94. doi: 10.2307/4127885

[4] Yu YY Zhang Y Feng J. The impact of long-term care insurance in supply side:Financial incentives and market entry. Econ Q. (2025) 2:293–309. doi: 10.13821/j.cnki.ceq.2025.02.02

[5] National Bureau of Statistics of China. (2023). Available online at: http://www.gov.cn/xinwen/2023-02/28/content.5743623.htm (Accessed February 28, 2023).

[6] Polanco B Oña A Sabariego C Pacheco Barzallo D. Chronic health conditions and their impact on the labor market. A cross-country comparison in Europe. SSM. (2024) 26:101666. doi: 10.1016/j.ssmph.2024.101666, PMID: 38616807 PMC11015523

[7] Riekhoff AJ Vaalavuo M. Health shocks and couples’ labor market participation: a turning point or stuck in the trajectory? Soc Sci Med. (2021) 276:113843. doi: 10.1016/j.socscimed.2021.113843 33756129

[8] Tong YF Liao YH. The effects of Chinese older adults health on labor participation. Chin J Popul Sci (2017) 6: 105–116+128.

[9] Ci QY Cheng YR. Family-centered, active aging: an analysis of family factors influencing labor participation among rural elderly populations. New Horizons from Tianfu. (2022) 6:9.74–86. doi: 10.3969/j.issn.1004-0633.2022.06.009

[10] Li Q Tan N. Review on the relationship between health and the older adults labor supply. J Univ Electr Sci Technol China (Soc Sci Ed). (2019) 3:38–47. doi: 10.14071/j.1008-8105(2018)-1057

[11] Campolieti M. Disability and the labor force participation of older men in Canada. Labour Econ. (2002) 3:405–32. doi: 10.1016/S0927-5371(02)00051-9

[12] Dwyer DS Mitchell OS. Health problems as determinants of retirement: are self-rated measures endogenous? J Health Econ. (1999) 18:173–93. doi: 10.1016/s0167-6296(98)00034-4, PMID: 10346352

[13] Kalwij A Vermeulen F. Health and labour force participation of older people in Europe: what do objective health indicators add to the analysis? Health Econ. (2008) 17:619–38. doi: 10.1002/hec.1285, PMID: 17935200

[14] Mcgarry K. Health and retirement:do changes in health affect retirement expectations? J Hum Resour. (2004) 3:624. doi: 10.3368/jhr.39.3.624

[15] Cai L Kalb G. Health status and labour force participation: evidence from Australia. Health Econ. (2009) 15:241–61. doi: 10.1002/hec.1053 16229055

[16] Benjamin D Beand TL Fan JZ. Ceaseless toil? Health and labor supply of the elderly in rural China. Ann Arbor: William Davidson Institute (2003).

[17] Feng Z. Economic activities in old age: Experience of the urban and rural elderly in contemporary China. Providence: Brown University (2002).

[18] Li Q Lei XY Zhao YH. The effect of health on the labor supply of mid-aged and older Chinese. China Econ Q. (2014) 3:917–38. doi: 10.13821/j.cnki.ceq.2014.03.003

[19] Wang YJ Jin ZJ Yuan Y. The consequences of health shocks on households: evidence from China. China Econ Rev. (2023) 79:101969. doi: 10.1016/j.chieco.2023.101969

[20] Marinaccio A Gariazzo C Taiano L Bonafede M Martini D D'Amario S . Climate change and occupational health and safety. Risk of injuries, productivity loss and the co-benefits perspective. Environ Res. (2025) 269:120844. doi: 10.1016/j.envres.2025.120844, PMID: 39832550

[21] Gong Y Tang SL Ni YC Dong HQ Ni GX. Association analysis of labor force participation and health service utilization among middle-aged and older adults. Health Econ Res. (2024) 6:44–8. doi: 10.14055/j.cnki.33-1056/f.2024.06.011

[22] Yang ZH Tursun M Wang YP. The impact of health shocks on agricultural labor supply of rural middle-aged and older adults: empirical analysis based on CHARLS data. China Rural Survey. (2015) 3:24–37. doi: 10.20074/j.cnki.11-3586/f.2015.03.003

[23] Cui YY Xu ZY Cai YG Xia F. The influence of health shock on poverty of middle-aged and older adult families. Health Econ Res. (2023) 4:43–7. doi: 10.14055/j.cnki.33-1056/f.2023.04.007

[24] Wang ZY Chen GY. The impact of health shock on labor supply of rural middle-aged and older adults: Effect evaluation and evidence of mechanism.. Northwest Popul. (2021) 3:23–37. doi: 10.15884/j.cnki.issn.1007-0672.2021.03.003

[25] Mete C Schultz TP Health and labor force participation of the elderly in Taiwan. New Haven: Yale University Economic Growth Center (2002).

[26] Andrew MJ Nigel R Francesca Z. Acute health shocks and labour market outcomes:evidence from the post crash era. Econ Hum Biol. (2020) 36:100811. doi: 10.1016/j.ehb.2019.100811 31521566

[27] Zhao HH. Research on the consumption structure of the older adults and the influencing factors of consumption upgrading. Adv Appl Math. (2022) 8:5213–28. doi: 10.12677/AAM.2022.118551

[28] Islam MK Kjerstad E Rydland HT. The chronically ill in the labour market-are they hierarchically sorted by education? Int J Equity Health. (2024) 23:66. doi: 10.1186/s12939-024-02148-w, PMID: 38528545 PMC11409748

[29] Davide G Francesco S Giovanni G Jacopo P Paolo B Simona R . Gender differences in healthcare utilization across Europe: evidence from the European health interview survey. Health Policy. (2025):105448. doi: 10.1016/j.healthpol.2025.105448 41022014

[30] Peng XM Yang TT. The impact of chronic diseases on labor participation among the older adults. J Hubei Univ Econ (Hum Soc Sci Ed). (2021) 6:69–73. doi: 10.3969/j.issn.1671-0975.2021.06.017

[31] Cai L Cong C. Effects of health and chronic diseases on labour force participation of older working-age Australians. Aust Econ Pap. (2009) 2:166–83. doi: 10.1111/j.1467-8454.2009.00365.x

[32] Pedron S Emmert-fees K Laxy Y Schwettmann L. The impact of diabetes on labour market participation: a systematic review of results and methods. BMC Public Health. (2019) 1:25. doi: 10.1186/s12889-018-6324-6 PMC6323654 30616606

[33] Heinesen E Kolodziejczyk C. Effects of breast and colorectal cancer on labour market outcomes-average effects and educational gradients. J Health Econ. (2013) 32:1028–42. doi: 10.1016/j.jhealeco.2013.08.004 24096321

[34] Choi J-W Choi J-W Kim H Yoo KB Park EC. Association between chronic disease and catastrophic health expenditure in Korea. BMC Public Health. (2015) 15:26. doi: 10.1186/s12913-014-0675-1, PMID: 25608983 PMC4307618

[35] Liu EP Zhang QL Feng Y. Older adult poverty risk due to chronic diseases: Theoretical mechanisms and empirical analysis. Insurance Stud. (2020) 11:63–78. doi: 10.13497/j.cnki.is.2020.11.005

[36] Abegunde DO Stanciole AE. The economic impact of chronic diseases: how do households respond to shocks? Evidence from Russia. Soc Sci Med. (2008) 66:2296–307. doi: 10.1016/j.socscimed.2008.01.041, PMID: 18329147

[37] Tan N Zhou X B. Does “endless labor” exist among rural elderly in China?- A study on the impact of age and health on labor supply time.Econ Rev (2013) 2:19–29. doi: 10.19361/j.er.2013.02.003

[38] Liu RP Liu M. The impact of pension security on labor participation among the older adults and regional variations. Sci Res Ageing. (2016) 11:31–42. doi: 10.3969/j.issn.2095-5898.2016.11.004

[39] Zhao M Wang XJ. The effect of pension on labor participation of the young old in China. Popul Res. (2022) 4:69–83. Available online at: https://rkyj.ruc.edu.cn/CN/Y2022/V46/I4/69

[40] Haider S Loughran D. Elderly labor supply: Work or play? Labor Supply Demand. (2001) 45064756. doi: 10.2139/ssrn.285981

[41] Li W Xiang Y. The correlation of labor force participation and environment support with the health level among older adults. Mod Prevent Med. (2016) 15:2777–2780+2784.

[42] Song YP Zhang GY Peng KY. Current levels and changing patterns of labor force participation among older adults at age 60-69 in China. Popul Res. (2024) 2:75–89.

[43] Lve MY Peng XZ Lu MH. The effect of Internet use on employment of the older adults. Econ Perspect. (2020) 10:77–91.

[44] Wang LQ Lei XY. Medical consumption and health status of rural older adults in China:The impact of the new cooperative medical scheme. J Nanjing Agricult Univ (Soc Sci Ed). (2011) 2:33–40. doi: 10.3969/j.issn.1671-7465.2011.02.007

[45] Fu HQ Yuan D Lei XY. Health status, medical insurance, and pre-existing moral hazard: empirical evidence from China’s new rural cooperative medical scheme. China Econ Q. (2017) 2:599–620. doi: 10.13821/j.cnki.ceq.2017.01.07

[46] Wang HF Yang F. Do chronic diseases affect the older adults’ attitude toward aging?—An empirical analysis based on China longitudinal aging social survey. South China Popul. (2021) 3:67–80. doi: 10.3969/j.issn.1004-1613.2021.03.006

[47] Zheng W Xie ZW Lve YJ Han X. The impact of chronic diseases on spouses’ labor market performance for middle-aged and older adults. Insurance Stud. (2023) 5:70–81. doi: 10.13497/j.cnki.is.2023.05.006

[48] Guan F Li MS. Health, public medical insurance and labor participation among the older adults. Hum Resources Dev China. (2023) 1:90–104. doi: 10.16471/j.cnki.11-2822/c.2023.1.007

[49] Li YH Liu H. Literature review on health human capital research. Product Res. (2009) 20:189–92. doi: 10.19374/j.cnki.14-1145/f.2009.20.073

[50] Zhou XF Chen T Zang WB. The impact of new rural cooperative medical scheme on labor supply of agricultural workers. China Econ Stud. (2020) 3:30–42. doi: 10.19365/j.issn1000-4181.2020.02.03

[51] Xu S. The impact of the new rural cooperative medical system on the agricultural labor supply of the middle and aged people in rural areas. J Ningxia Univ (Soc Sci Ed). (2019) 6:125–31. doi: 10.27756/d.cnki.gzjlx.2019.000268

[52] Wang YY, Zeng YB Fang Y. A study on the effect of labor participation on the health of retired older adults in China.Chin J Health Policy (2021) 1:59–65. doi: 10.3969/j.issn.1674-2982.2021.01.009

[53] Jménez-Martin S. Strike outcomes and wage settlements in Spain. Labour. (2006) 4:673–98. doi: 10.1111/j.1467-9914.2006.00360.x

[54] Mo JL Song Z. An analysis of labor supply of middle-aged and older adults in China. Popul Econ. (2012) 4:55–63.

[55] Li JX Xia CC. The diseases transition in Chinese older adults: Traditional and modern pattern. Popul Dev. (2019) 4:94–105.

[56] Cole D. Occupational health hazards of agriculture: understanding the links between agriculture and health. Washington, DC: International Food Policy Research Institute (2006).

[57] Trevisan E Zantomio F. The impact of acute health shocks on the labour supply of older workers:evidence from sixteen European countries. Labour Econ. (2016) 43:171–85. doi: 10.1016/j.labeco.2016.04.002

[58] Schultz T P Tansel Aysit. Measurement of returns to adult health: Morbidity effects on wage rates in cote d'Ivoire and Ghana (English). Living standards measurement study (LSMS) working paper; no. LSM 95 Washington, DC: The World Bank. Available online at: http://documents.worldbank.org/curated/en/862031468770671403 (Accessed April 30, 1993).

[59] Li Q Zhao R Zhang TL. How does How does becoming widowed affect the rural older adults’ health? Based on the data of CHARLS. Nankai Econ Stud. (2022) 2:157–76. doi: 10.14116/j.nkes.2022.02.010

[60] Vere JP. Social security and older adults labor supply: evidence from the health and retirement study. Labour Econ. (2011) 5:676–86. doi: 10.1016/J.LABECO.2011.02.001

[61] Kaushal N. How public pension affects elderly labor supply and well-being: evidence from India. World Dev. (2014) 56:214–25. doi: 10.1016/j.worlddev.2013.10.029

[62] Zhang CC. Pension income and labor supply among rural older adults populations: an analysis based on regression discontinuity design. World Econ Papers. (2015) 6:76–89.

[63] Pei JS Jiao M. Research on the substitute effect of social insurance on the children of -rural left-behind older adults-Based on CHARLS’s micro data. J Hebei Univ (Philos Soc Sci). (2020) 5:125–35. doi: 10.3969/j.issn.1005-6378.2020.05.015

[64] Gu X Zhou Y. New rural cooperative medical scheme:adverse selection and moral hazard. Shanghai Finance. (2021) 2:15–25. doi: 10.13910/j.cnki.shjr.2021.02.002

[65] Zhang CC Wei X Huang W. Interactions among social security programs: the crowding out of medical insurance by the new rural social pension insurance. China Econ Q. (2023) 3:860–75. doi: 10.13821/j.cnki.ceq.2023.03.03

